# Improved molecular toolkit for cAMP studies in live cells

**DOI:** 10.1186/1756-0500-4-241

**Published:** 2011-07-20

**Authors:** Kwan Pyo Hong, Nicholas C Spitzer, Xavier Nicol

**Affiliations:** 1 Neurobiology Section, Division of Biological Sciences, Kavli Institute for Brain and Mind, University of California, San Diego, La Jolla, CA 92093, USA; 2 INSERM, U839, Université Paris 6, Institut du Fer à Moulin, 17 rue du Fer à Moulin, 75005 Paris, France

## Abstract

**Background:**

cAMP is a ubiquitous second messenger involved in a wide spectrum of cellular processes including gene transcription, cell proliferation, and axonal pathfinding. Precise spatiotemporal manipulation and monitoring in live cells are crucial for investigation of cAMP-dependent pathways, but existing tools have several limitations.

**Findings:**

We have improved the suitability of cAMP manipulating and monitoring tools for live cell imaging. We attached a red fluorescent tag to photoactivated adenylyl cyclase (PACα) that enables reliable visualization of this optogenetic tool for cAMP manipulation in target cells independently of its photoactivation. We show that replacement of CFP/YFP FRET pair with GFP/mCherry in the Epac2-camps FRET probe reduces photobleaching and stabilizes the noise level during imaging experiments.

**Conclusions:**

The modifications of PACα and Epac2-camps enhance these tools for *in vitro *cAMP studies in cultured living cells and *in vivo *studies in live animals in a wide range of experiments, and particularly for long term time-lapse imaging.

## Background

cAMP is a major cellular second messenger that activates and integrates multiple intracellular signaling pathways and modulates a large range of cellular processes, including gene transcription [1], cell adhesion and migration [2], and axonal growth and pathfinding [3]. cAMP studies rely on methods to manipulate and monitor cAMP concentrations in live cells. Existing tools have been very useful in identifying cAMP-dependent cellular processes, but have some limitations when it comes to understanding cAMP dynamics and localization in living cells. Forskolin and 3-isobutyl-1-methylxanthine (IBMX) are powerful pharmacological compounds enabling the generation of sustained elevations of cAMP. Forskolin directly stimulates most transmembrane adenylyl cyclases [4] and IBMX inhibits cAMP hydrolysis by phosphodiesterases. Recently, the use of photoactivated adenylyl cyclase alpha (PACα) from the flagellate *Euglena gracilis*, synthesizing cAMP in response to blue light, has allowed precise spatiotemporal manipulation of cAMP [5]. It has been attached to GFP for visualization in live cells [6]. However, the excitation wavelength of this visible reporter overlaps with the excitation spectrum of PACα, making it difficult to use this fusion construct for independent PACα excitation and reporter imaging.

Monitoring cAMP in live cells has been made possible by the use of FRET probes [7-10]. Epac2-camps is a cAMP indicator that is widely used to monitor cAMP [10] and has been recently improved with a mutation increasing its affinity for cAMP [11]. However, fast photobleaching of the commonly used CFP/YFP FRET pair limits its use in live cell imaging experiments over extended periods of time because the signal-to-noise ratio decreases progressively. The GFP/mCherry FRET pair has been successfully used for cAMP sensors [12], but its photostability and signal-to-noise ratio have not been assessed.

## Results

### Independent excitation of PACα and mCherry in living cells and live animals

**Figure 1:**
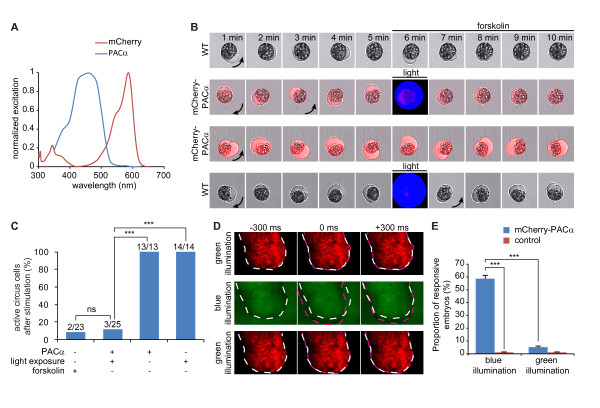
**Characteristics of mCherry-PACα**. (A) PACα and mCherry have distinct excitation spectra, predicting independent excitation. (B, C) Sequences of merged panels of transmitted light images, mCherry fluorescence images (red), and 488 nm PACα excitation (blue). The transmission image is omitted during the 488 nm excitation because of saturation of the image (6 min, 2^nd ^and 4^th ^rows). Both bath application of 10 μM forskolin (1^st ^row) and one-minute photoactivation of PACα (2^nd ^row) induce arrest of circumferential movements of circus cells' cytoplasm. In contrast, excitation of mCherry alone (3^rd ^row) or blue light illumination of cells lacking PACα (4^th ^row) does not affect circus cells' movements. Arrows indicate the direction of the circus movement in the first image of each row and each time the direction switches. Cells were split in 4 quadrants and were scored as moving when a blob of cytoplasm was seen in all the quadrants during the 4 minutes following stimulation. ≥ 13 cells were scored for each condition. (D, E) mCherry-PACα-injected embryos twitch when illuminated with blue light to excite PACα (2^nd ^row), but not when exposed to green light to excite mCherry (1^st ^and 3^rd ^row). Dashed white line marks the initial position of the head of the embryo (-300 ms). Dashed magenta line indicates the head position during (0 ms) and following (+300 ms) the twitch. Embryos that do not express mCherry-PACα are insensitive to both blue and green light. Images were extracted from the movie shown in Additional File 2. Images were chosen 300 ms before (-300 ms), during (0 ms) and 300 ms after (+300 ms) the embryo twitches under blue illumination (BP 470/40 filter). Embryos were immobile under green illumination (BP 560/40 filter), and the 600 ms portions of the recording was chosen randomly (1^st ^and 3^rd ^row). Embryos were scored as responsive when they were not completely immobile during the illumination period. ≥ 35 embryos were scored for each condition. Error bars, sem. *** p < 0.001. C, Chi Square test. E, ANOVA.

The red fluorescent protein mCherry was expected to be an appropriate tag for PACα since its excitation wavelength in the green range of visible light [13] is distinct from PACα excitation by blue and UV light [14] (Figure 1A). A mCherry-PACα fusion protein was generated using a mutant of PACα (R330A) that has a limited adenylyl cyclase activity in the dark (G. Nagel, personal communication). mCherry was attached to the N-terminus of PACα (linker: SGLRSRAQASNSAVDGTA). The fluorescence of mCherry and light-dependent cAMP synthesis of PACα appear unaffected in the fusion product. mRNA coding for mCherry-PACα was transcribed using the mMessage Ultra kit (Ambion). 1 to 3 ng of mRNA were injected in both blastomeres of 2-cell stage *Xenopus laevis *embryos, which were incubated in the dark for 24 hr at 23°C. Dissociated cells from stage 21 embryo neural tubes were plated onto plastic dishes and kept in the dark for 2 hr. Cultures from injected but not control animals were fluorescent when illuminated at 561 nm on a Leica SP5 confocal microscope, revealing the expression of mCherry (Figure 1B). We then developed a bioassay to assess the function of this construct. Circus cells exhibit circular movement of the plasma membrane in cultures from *Xenopus *neural tubes [15]. Application of 10 μM forskolin blocked the circus movements of these cells (Figure 1B and 1C), demonstrating that a high concentration of cAMP prevents these plasma membrane movements. We next tested the light-dependent cyclase activity of mCherry-PACα by illuminating mCherry-positive cells at 488 nm. A 1-minute exposure abolished membrane movements. In contrast, mCherry excitation did not affect circus movements and mCherry-negative cells did not exhibit plasma membrane movement arrest following stimulation by either wavelength (Figure 1B and 1C and see Additional file 1). We conclude that mCherry-PACα combines the features of mCherry for cell identification and PACα for light-sensitive cyclase activity, and that mCherry and PACα excitation wavelengths are exclusive from each other.

To verify that this construct can be used *in vivo*, *Xenopus *embryos injected with mCherry-PACα mRNA were illuminated with green and blue light in alternation using a fluorescence dissecting microscope with GFP and Texas-red filter cubes. Blue light illumination (excitation filter: BP 470/40) induced embryos to twitch, whereas they remain completely immobile under green light illumination (excitation filter: BP 560/40) (Figure 1D and 1E and see Additional file 2). Uninjected embryos did not exhibit light-induced twitching. Excitation of mCherry did not affect embryos' behavior, confirming the spectral compatibility of mCherry and PACα.

### Reduced photobleaching of Epac2-camps using the GFP/mCherry FRET pair

**Figure 2:**
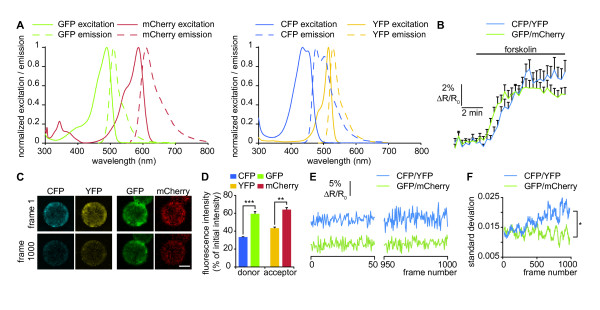
**Reduced noise level of pm-Epac2-camps-GFP/mCherry compared to pm-Epac2-camps-CFP/YFP**. (A) Normalized excitation (solid lines) and emission (dashed lines) of GFP, mCherry, CFP and YFP. Emission spectra of CFP and YFP largely overlap, making FRET measurement difficult. The large separation of GFP and mCherry emission spectra significantly reduces crosstalk between acceptor and donor. (B) ΔR/R_0 _was computed as (R-R_0_)/R_0 _where R is donor:acceptor ratio and R_0 _the mean value of this ratio before forskolin stimulation. ΔR/R_0 _of both pm-Epac2-camps-CFP/YFP and pm-Epac2-camps-GFP/mCherry increase after 10 μM forskolin application. The lag between the pm-Epac2-camps-GFP/mCherry and pm-Epac2-camps-CFP/YFP signals falls under the precision of the perfusion system and is unlikely to reflect a difference between the sensors. No correction factor was applied. ≥ 15 cells were scored for each condition. (C) Acceptor and donor emission of pm-Epac2-camps-CFP/YFP and pm-Epac2-camps-GFP/mCherry before and after 1000 frame acquisitions (excitation with a 442 nm laser line). Photobleaching is less pronounced for pm-Epac2-camps-GFP/mCherry. (D) Fluorescence intensity of acceptors and donors of pm-Epac2-camps-CFP/YFP and pm-Epac2-camps-GFP/mCherry after 1000 frames, expressed as a fraction of the fluorescence intensity of the first frame. ** p < 0.01, *** p < 0.001. (E) Noise levels for pm-Epac2-camps-CFP/YFP and pm-Epac2-camps-GFP/mCherry are equivalent for the first 50 frames. After 950 frames, only the noise level for pm-Epac2-camps-CFP/YFP has increased. Data are normalized to the value at the beginning of the recording as in B (ΔR/R_0_). (F) The standard deviation of ΔR/R_0 _over 40 frames, indicating the noise level, shows a progressive increase for pm-Epac2-camps-CFP/YFP but not for pm-Epac2-camps-GFP/mCherry. * p < 0.05 from frame 819 to 1000. C-F, 5 cells per condition. Error bars, sem. D, Kruskal-Wallis test. F, Mann-Whitney U test.

In addition to the improvement of PACα to manipulate cAMP, we modified the FRET pair of the existing Epac2-camps cAMP sensor to improve the stability of its signal-to-noise ratio over longer periods of time. We replaced the CFP/YFP pair by GFP/mCherry, with the same linkers as in the original probe [10]. mCherry and GFP have a low photobleaching rate [16] and constitute an efficient FRET pair [12] with smaller overlap between the emission spectra of the acceptor and the donor (Figure 2A). In addition, we included a mutation in the cAMP binding domain (K405E) that has been shown to reduce the Kd of the probe from 900 nM to 300 nM [11]. To demonstrate the improvement of the sensor, mRNA coding for membrane-targeted Epac2-camps including either CFP/YFP (pm-Epac2-camps-CFP/YFP) or GFP/mCherry (pm-Epac2-camps-GFP/mCherry) was transcribed and expressed in *Xenopus laevis *neural tube cultures as described for mCherry-PACα. Cultures were continuously superfused and three images per minute were acquired with 442 nm laser excitation and filters adapted for each FRET pair (CFP: 460 nm-500 nm; YFP: 525 nm-580 nm; GFP: 480 nm-550 nm; mCherry: 590 nm-700 nm). 10 μM forskolin induced a similar FRET ratio increase for pm-Epac2-camps-CFP/YFP and pm-Epac2-camps-GFP/mCherry, revealing an appropriate FRET response to cAMP elevation for both probes (Figure 2B). To assess the resistance to photobleaching, cells were continuously excited at 442 nm. CFP and YFP intensity decreased rapidly whereas GFP and mCherry showed reduced photobleaching (Figure 2C and 2D). Although 442 nm is not at the peak of excitation of GFP, a forskolin-induced increase of the GFP:mCherry ratio can be detected using this excitation wavelength (Figure 2B). Exciting GFP with a 442 nm laser line has the advantage of minimizing the direct excitation of the acceptor (mCherry) that perturbs FRET measurements when longer excitation wavelengths are used. To assess the noise level, we monitored the standard deviation of CFP:YFP and GFP:mCherry FRET ratio changes over 40 frames during the 1000 frames imaging period. The standard deviation of pm-Epac2-camps-GFP/mCherry was stable throughout the measurement period, while the standard deviation of pm-Epac2-camps-CFP/YFP increased over time (Figure 2E and 2F). The progressive increase of the noise level over time may be due to the increased relative contribution of the noise to the fluorescence measurement of each channel: the fluorescence intensity decreases because of photobleaching, but the noise level is not affected. Consequently, the noise level of the donor:acceptor ratio is higher after photobleaching.

## Conclusion

In summary, we have generated an improved toolkit for cAMP studies. mCherry-PACα allows spatiotemporal control of cAMP in living cells after identification of cells expressing it. Localized illumination of a cell is likely to increase cAMP concentration locally. Further development of PACα may include its targeting to subcellular compartments to go beyond the limit of precision of optical stimulation and achieve cAMP manipulation bearing closer resemblance to physiological signals. It would be useful to ensure for each experimental condition that the cyclase activity of PACα in the dark does not affect intracellular signaling [5,17]. To limit cAMP synthesis without light exposure, we used a mutated PACα (R330A) that has a limited cyclase activity in the dark (G. Nagel, personal communication). This was enough to avoid perturbation of circus cells movement by the cyclase activity of PACα in the dark. In case an extremely low dark activity is needed, bPAC, a bacterial light-sensitive adenylyl cyclase, could be used at the cost of less stringent temporal control of cAMP signaling [17].

pm-Epac2-camps-GFP/mCherry has greater photostability than pm-Epac2-camps-CFP/YFP and a lower noise level after extended periods of imaging. However GFP and mCherry make a less effective FRET pair than CFP and YFP, and its use may be beneficial only for FRET experiments requiring an extended period of imaging. Versions of CFP and YFP (mTurquoise and Venus respectively) with increased photostability are now available and make an efficient FRET pair for cAMP sensors [18]. Testing its noise level stability would allow comparison of the behaviour of GFP/mCherry and mTurquoise/Venus as FRET pairs for prolonged experiments. The sensor described here has the advantage over the mTurquoise/Venus probe [18] of sensitivity to lower concentrations of cAMP, because it is an Epac2-based sensor including a mutation that reduces its Kd [11]. It would be useful to compare it to the Epac1-based sensor using mTurquoise/Venus, with the higher Kd for cAMP [9,10,18].

The use of both tools in the same cell is not yet possible due to the overlap of excitation wavelengths, but further improvements may include the shift of PACα excitation towards the UV to avoid wavelength conflict with the FRET probe excitation, in combination with the switch of the mCherry tag to a longer wavelength fluorescent protein such as mKate to avoid the overlap of emission between the PACα tag and the FRET acceptor.

## Competing interests

The authors declare that they have no competing interests.

## Authors' contributions

KPH and XN carried out the experiments. NCS and XN conceived the experimental design. KPH, NCS and XN wrote the manuscript. All authors have read and approved the final manuscript.

## Supplementary Material

Additional file 1 **Photoactivation of PACα mimics forskolin-induced arrest of circus cells in culture**. In circus cells in cultures from the *X. laevis *neural tube, blebs of plasma membrane propagate around the cell circumference with a period of several minutes (Olson, 1996). Forskolin stimulation of endogenous adenylyl cyclases in control cells arrests this blebbing movement (top left panel). Blue light (488 nm) irradiation of mCherry-PACα-expressing cells mimics the effect of forskolin stimulation (top right panel). In contrast, blebbing movements are not affected in non-blue light-irradiated mCherry-PACα-expressing cells (bottom left panel) or in control cells illuminated at 488 nm (bottom right panel). Click here for file

Additional file 2 **Photoactivation of PACα induces *X. laevis *embryo twitching**. mCherry-PACα-injected embryos twitch when illuminated with blue light to excite PACα, but not when exposed to green light to excite mCherry. Click here for file
